# Integrative emphases on intimate, intrinsic propensity/ pathological processes–causes of self recovery limits and also, subtle related targets for neuroprotection/ pleiotropicity/ multimodal actions, by accessible therapeutic approaches–in spinal cord injuries

**Published:** 2010-08-25

**Authors:** G Onose, M Haras, D Mureşanu, C Giuglea, D Chendreanu

**Affiliations:** * >‘Carol Davila’ University of Medicine and Pharmacy, BucharestRomania; ** >‘Bagdasar–Arseni’ Teaching Emergency Hospital, BucharestRomania; *** >Neurological Clinic of ‘Iuliu Haţieganu’ University of Medicine and Pharmacy, Cluj– NapocaRomania

## Abstract

**Background**: The last two decades have come up with some important progresses in the genetic, immune, histochemical and bio (nano)–technological domains, that have provided new insight into cellular/ molecular mechanisms, occurring in the central nervous system (CNS) – including in spinal cord – injuries.

**Methods**: In previous works, emerging from our theoretical and practical endeavors in the field, we have thoroughly described the principal intimate propensity and the pathophysiological processes – representing intrinsic limitations for self–recovery after SCI, and, at the same time, subtle targets for neuroprotection/ recovery – and reviewed the main related worldwide–published reports. The aim of this paper is to emphasize the connections between such main aspects and some feasible integrative solutions, including the ones for clinical practice.

**Results**: Consequently, we stress upon some therapeutic suggestions regarding this subject matter by systematizing the most up to date and efficient ones – obviously, within major limits, according to the very low capacities of CNS/ spinal cord (SC) to post–injury self preserve and recover. Moreover, we also talk about accessible drugs, respectively those being already in clinical use (but at present, mainly used to treat other conditions, including the neurological ones) and hence, with relatively well known, determined effects and/or respectively, restrictions.

**Discussions**: The recent advances in the knowledge on the basic components of the afore mentioned CNS/ SC propensity for self destroying and inefficient endogenous repair mechanisms in the actual new context, will hopefully be, from now on, more effectively correlated with revolutionary – mostly still experimental – treatments, especially by using stem cells within tissue engineering, including, if needed, more advanced/ courageous approaches, based on somatic cell nuclear transfer (SCNT).

**Conclusions**: This paper contains the scientific motivated highlighting of some already available drugs, ‘neuroprotective’ (and not only) properties too, which enable practitioners with (although not yet capable to cure – but anyway) more efficient therapeutic means, to approach the extremely difficult and still painfully disappointing domain, of spinal cord injury (SCI).

## Background. SCI current data. Actual context

The closest to reality and updated statistics data, including the data with respect to SCI, are to be found in the United States of America (USA – that is why, below we present a few more details, mainly of this provenance). Yet, even there, probably because it refers to a very long lasting unsolved problem, there have not been any overall incidence studies on SCI since the 1970s and hence, it cannot be known precisely whether the incidence has changed lately [1]. 

Anyway, an estimated rate of 2.5 million people live with SCI, with more than 130,000 new injuries reported each year worldwide [2]. 

There were between 229,000 and 306,000 (estimated to be approximately 250,000 – 259,000) persons, alive, in USA, in 2008, having SCI (52% of the spinal cord injured individuals are considered paraplegic and 47% quadriplegic), with an incidence (see the above–mentioned comments) of approximately 11,000 – 12,000 (40 cases per million population – 0.04 per thousand) additional people, yearly. Until 1979, the average age at injury was of 28.7 years, as the median age of the general population of the United States (likewise, more or less, practically, worldwide) has increased with approximately 8 years, from the mid–1970's. Moreover, the average age at injury has also steadily increased accordingly (31 years somewhere in the first part of a three decades period) over time. Since 2005, the average age at injury has been of 40.2 years. Currently, 80.9 – 82 % of the SCI occurred among males [3,4]. 

It is appreciated that in the member states of the Council of Europe there are at least 330,000 subjects with about 11,000 new cases every year. Approximately 40% to 50% of these injuries are the result of road accidents and mostly occur at a young age [5].

Statistics on chronic patients with SCI in Romania, as in most other countries, are very difficult to compile and update (due to practical difficulties in maintaining a central database of case histories). However, we succeeded to quite satisfactory assess (just) their incidence (and only to approximate prevalence). Hence, according to our last query of the Diagnosis Related Groups (DRG – the Australian classification) the National database of inpatient care [6], we have found, in 2008, 419 new cases of hospitalized patients with SCI, accounting for an incidence of 0.0194 per thousand. Subsequently, we could estimate the total number of such patients in Romania to be around 15,000–20,000 +/– a few thousands. The average age was of 41.4 years [7].

**Figure 1:**
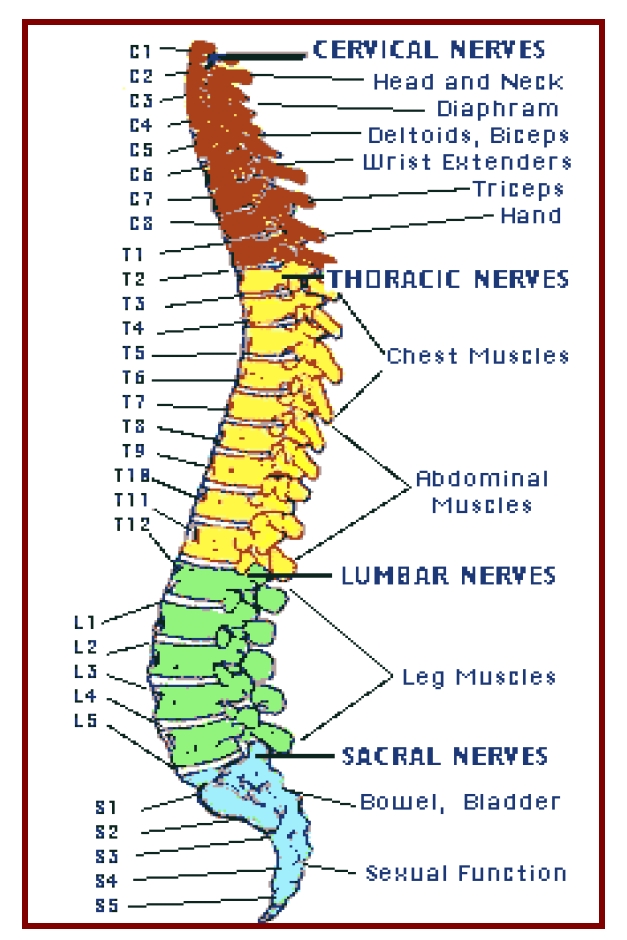
The spinal column and the essential muscles and somatic/ visceral functions depending on the spinal cord and nerves, at corresponding metameric levels – by [8]

SCI usually generate severe and rather permanently, loss or impairment of basic functions, such as: voluntary/ active mobility, sensitivity, micturition and/or defecation control, erection/ ejaculation/ fertility [9–12]; therefore, they are, in most of the cases devastating, especially because they are frequently irreversible.

On long–term or (quasi)continuously, they are usually associated with serious co–morbidities, emerging from: tissue distrophicity (mainly pressure sores), urinary tract infections (UTI – chronic/ recurrent), metabolic and/or (including related) circulation disturbances: of blood pressure [13], respectively of the venous–lymphatic flow in lower limbs especially the anti–gravitational one [14].

SCI can be divided into two main types of lesion/ functional damages, i.e. complete and incomplete.

Complete means there is no function/ control below the neurological level of injury: no sensations and no voluntary movement is preserved (including) in the sacral segments S4,5.

Incomplete SCI means there is some functioning below the injury level. Such a person may feel parts of the body that cannot be moved, including the sacral segments S4,5 (type B on the American Spinal Injury Association Impairment  Scale – AIS B) or have some active motility/ functional control preserved too (AIS C, D) [9], on both sides of the body. In fact, the segments at which the normal function is found, often differ according to the side of the body and in terms of sensory vs. motor testing; (thus, up to four different segments may be identified in the determination of the neurological level, i.e.: R–sensory, L– sensory, R–motor, L–motor. In such cases, it is strongly recommended that each of these segments is separately recorded). When a single (average /global/ synthetic) ‘level’ is used, the term Sensory Level refers to the most caudal segment of the spinal cord with (quasi) normal sensory function on both sides of the body, similar to the Motor Level [10].

The effects of the SCI essentially depend on the spine/(al) cord topographic level of the injury, too – i.e.: paraplegia, after thoracic and/or lumbar and respectively, tetraplegia (from all points of view: generally biological – having also a life threatening  potential, especially during the acute and sub–acute phases post injury – and functional, including: self care/ autonomy, family relations, work and social activity/ participation, quadriplegia is by far more serious than paraplegia, being in fact, one of the most severe and invalidating sufferings within the human pathology), following cervical,  SCI.

Usually, post injury, no significant regenerative processes naturally occur in the CNS, including the SC: conversely, there are strong intrinsic hampering; (para) physiological and pathological processes that prevent spontaneous recovery, thus explaining the still poor therapeutic/ rehabilitative outcomes, obtained in the approach of these conditions.

Actually, the SCI treatment is generally limited to surgical intervention – decompression, drainage and stabilization – and to complex (supportive and assistive) care, intricated with long–term rehabilitation programs and respectively, follow–up. 

Still, after the 1990's, the advances in medical, surgical and rehabilitative technologies, significantly improved the length and quality of life for people with SCI. Because there is no cure for SCI and its devastating sequels, many researchers are addressing this matter and subsequently, there have been progresses in the laboratory. However, none of them succeeded, to produce radically effective healing outcomes expected/ long awaited by the patients, until now, i.e. to spectacularly improve the neurological/ functional outcome. 

Currently, as already mentioned, there are no effective therapies for SCI, except for the partially prevention of further, extending post injury, damages: neuroprotection/ pleiotropic (a quite rare property, specific to neurotrophic factors – fulfilling both, neuroprotection and neuroplasticity stimulation) and – possibly, at best – for some multimodal (i.e. including neurotrophicity and neuro/ synaptogenesis), effects [1,15, 16].

 At present, the Obama Administration of the USA has compared a radically different opinion regarding the new biotechnological researches to the former one, i.e. a very supportive one. Hence, aside a very generous unprecedented high budget allocated for research and development [17] it has recently also changed the composition of the American Bioethics Commission – that will move beyond the issues that consumed the previous panels, such as stem cells and (therapeutic) cloning. [18].

Hence, almost one year ago, the President of the USA, Barack Obama, officially declared – and acted this way: 

‘Today, with the Executive Order I am about to sign, we will bring the change that so many scientists and researchers, doctors and innovators, patients and loved ones have hoped for, and fought for, these past eight years: we will lift the ban on federal funding for promising embryonic stem cell research. We will vigorously support scientists who pursue this research. And, we will aim for America to lead the world in the discoveries it may yield one day.At this moment, the full promise of stem cell research remains unknown, and it should not be overstated. But, scientists believe these tiny cells may have the potential to help us understand, and possibly cure, some of our most devastating diseases and conditions. To regenerate a severed spinal cord and lift someone from a wheelchair. To spur insulin production and spare a child from a lifetime of needles. To treat Parkinson's, cancer, heart disease and others that affect millions of Americans and the people who love them. … However, in recent years, when it has come to stem cell research, rather than furthering discovery, our government has forced what I believe it is a false choice between science and moral values. In this case, I believe the two are not inconsistent. As a person of faith, I believe we are called to care for each other and work to ease human suffering. I believe we have been given the capacity and will to pursue this research – and the humanity and conscience to do so responsibly’ [19].

Therefore, a most important, general – at least intermediate – message for all post SCI para– and tetra–plegics to take home, is to remain, according to the well–known – old but more and more actual World Health Organization (WHO)'s definition of a complete state of health – as healthy as possible (physically, mentally, socially) and active: in family, professionally and ad vocationally. 

## Integrative emphases regarding limits, detrimental pathways and related targets for neuroprotection/ recovery, in SCI

Herein we (re)emphasize that there are morpho–functional, inner, restrictions of the CNS/ SC's post injury self–repair ‘skills’, within a reactive behavior prone to self–augmenting pathological, destructive processes, which result in vicious circles, in further limitations (mainly: neurons, lacking centrosoms, cannot reproduce/ regenerate; there are pre–formate pathways, which, from yet unknown reasons, exert active opposition to axonal re–growth) and that these intrinsic, misfortunate, ‘brakes’ to self–recovery and respectively, propensity to detrimental evolutive pathways, are the principal actual targets for neuroprotective/ pleiotropic (possibly even multimodal) therapies, in order to reduce, as much as possible the ‘secondary injuriy(es)/ lesion(s)/ damage(s) processes'/ events' cascade’ and consequently, to obtain better further recovery/ rehabilitation [1,15].

CNS injuries may be divided into two main categories: primary – which occur (mainly) at the moment of a trauma – and secondary ones, that develop after the initial injury. 

The mechanisms of ‘primary injury’ are not yet completely understood; however, (including relatively) intense – which is frequently the case – SC contusion (not physical trans–section) produces, from the morphological point of view, a central hemorrhagic necrosis – the ‘epicenter’ of the initial SC lesion – surrounded by disrupted and respectively – farther – surviving axons, with centrifugal distribution [1]

The cascade of pathophysiological events leading to secondary damage, is quite similar in traumatic (and also ischemic) injuries, of both the brain and the SC and therefore, neuroprotective/ pleiotropic (possibly even multimodal) therapies are, in many respects, resembling [1,15]

Still, there are structural non–similarities, resulting in different responses to traumatic insults between SCI and TBI: SC – crucial tracts are placed closer to the dural surface (superficial) . As far as the brain is concerned – deeper structures are often the critical ones.

Secondary damages develop after the initial/ primary injury – as a consequence of a complex, rather specific pathophysiological events ‘cascade’ to CNS/ SC, and produced within vicious circles – in the affected area and its neighborhood – effects that may continue for a long time [1,15].

**Figure 2:**
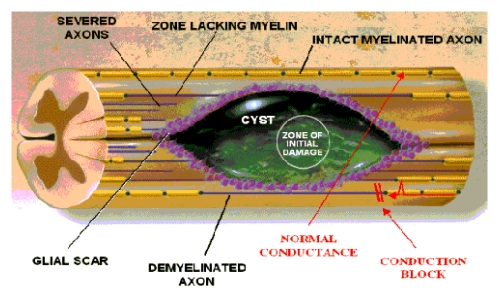
Late post traumatic SC cyst/ syrinx, ‘based on’ acute/ initial central hemorrhagic necrosis - by [22]

The secondary injury processes, briefly consist of: excessive synthesis of nitric oxide (NO), harmful metabolic hyperactivity – including/ conjugated with (mainly) phosphodiesterase 4 mediated myelin–associated Neurite (Out)growth Inhibitor (protein, called NOGO–A). The indirect stimulation presupposes the activation of its receptor results in the depletion of a cellular cyclic adenosine monophosphate (cAMP), i.e. in dramatically disturbance/ depletion of cell energetics – and oxidative stress, microglia activation and microglia–related cytokine unbalance, immune shifts and local inflammation, (including through prostaglandins' chemotactism – enhancing local inflammation and also, secondary, local/ regional vasoconstriction/ ischemia) the alteration of the regional (micro) circulation – including the cerebro–spinal fluid, with blood–brain (haemato–nevraxial) barrier dysfunction– electrolyte disturbances – resulting in nervous tissue swelling, including massive edema – followed (because of suddenly installed osmolysis) by cell induced necrosis and “delayed mechanisms of cell death” – apoptosis and apoptosis–like processes – lead, in sum – cumulated with the primary ones – to severe/ (most of them) irreversible, consequences: neuronal necroses and apoptoses, demyelization, scar and cyst formation, disruption of morpho–functional nerve pathways/ disconnection (Figure 2), deliverance of ‘specialized’ molecules in active, pre–formated, block of axons' re–growth [1,15,20,21].

The secondary events' ‘cascade’ entail an extremely complex and extended reaction, practically of the entire affected body: from its gene level to the ‘macroscopic’/ clinical, one. 

So, in order to obtain efficient neuroprotective/ pleiotropic/ multimodal therapeutic results and thus – maybe, even – a future cure, as many as possible (eventually – the whole picture) of its intimate, necessary to be approached, items/ targets had to be known: the ones to be counteracted, the others to be promoted. 

Hence, many molecules expressed by heterogonous glial and neural subpopulations of cells have been identified, which are directly or indirectly critical  for tissue damaging/ sparing/ re–growth inhibiting/ promoting, angiogenesis, neural plasticity, and also, various substances/ energy vectors with neuroprotective/ biotrophic and/or regenerative properties, as mentioned in previous articles[1,15]. 

More specifically, the secondary injury processes' cascade, at cell/ molecular level, mainly includes about 18 pathways (in the near future their type and consequently, their number, could change, as the researches in this fascinating field are intensely progressing). According to the state–of–the–art, we have already systematized them into a detailed presentation [15].

Yet, for a better understanding – and this is one of the aims of the present paper – some further specifications/ details are necessary, as from objective reasons, concerning previous related works, there have been (and will always be) editorial space constraints. 

Hence, so as to identify and achieve integrative connections with the clinical practice as efficient as possible, a stress has to be made upon some of the principal – especially by their quantitative amplitude/ extension and subsequently, contribution to the overall post SCI damage –  intimate pathological processes involved, i.e. the: oxidative stress, based on an imbalance between a very high increase of oxygen reactive species (ORS) production (including serum iron/ labile Fe pool, mediated – Figure 6) and down regulation of antioxidant systems’ amount/ effectiveness; disimmune and diselectrolitic mediated tissue disorders, including the ones of the local/ regional microcirculation – resulting in ischemia and edema (the latter leading, by osmolysis and cell passively die off to necrosis – thus to cell loss – augmented by conjugated destroying actions of ORS, endogenous enzymes, with ”digestive” actions on the main kinds of biological membranes’ compounds (protean and lipid); excess of neurotransmitters (more complex – especially, glutamate – and also some chemically simple ones – but equally dangerous – such as calcium ions); last but not least – different complicated pathways/ processes of apoptosis [15]. 

More detailed, main ORS are the following: superoxide anion (O_2_–), hydroxyl anion (OH–), hydrogen peroxide (H_2_O_2_) and peroxinitrite (ONOO–) . 

Critical concentrations of NO, together with – by increased Ca^2+^ cellular influx – activation of neuronal nitric oxide–synthase (nNOS), – again – in the presence of Ca^2+^ (absorbed at the mitochondria level) convert NO into ONOO–: one of the most ‘active’/ horrid  ORS. Moreover, the intense toxicity, inflammation (by alterating mechanisms of gene transcriptional factors type – resulting in stimulation of pro–inflammatory cytokines' production) triggering capacity and pro–apoptotic actions of ORS [15], are known. 

Additionally, other by–products of the post SCI secondary injury pathways – also dreadful endogenous tissue ”poisons” – are the unsaturated aldehydes (end compounds of lipid peroxidation, that also result in higher quantities, during oxidative stress), mainly: Acrolein  [2–propenal/ Acr(yl)aldehyde (C3H4O) – highly electrophilic, is one potential mediator of secondary damage, being capable of diffusing from compressed tissue to the adjacent, otherwise uninjured, ones – [23] and 4–hydroxynonenal [trans–4–hydroxy–2–nonenal or 4–HNE (C_9_H_16_O_2_) – that has been hypothesized to play a key role in cell signal transduction in (thus affecting) a variety of pathways (from cell cycle – in a concentration–dependent manner – to cellular transfection and adhesion events), interfering – including – with induced by oxidative stress apoptosis (through its regulation of mediated signaling) by a crucial (neuro)protective antioxidant endogenous enzyme system: of glutathione's/Glutathione S–transferases (GSTs) – [24]. 

**Figure 3:**
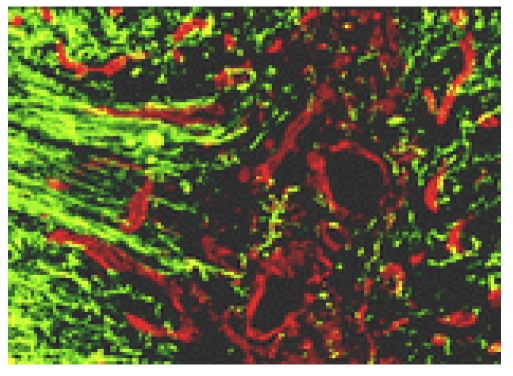
Glial/ fibrous collagen, scar formation: green–nerves; red–scar – by [27]

Aside their direct and indirect toxicity, all these by–products, including components of the dead or dying cells (injury to the spinal cord results, histological,  in damaged: neurons, glial cells, blood vessels and meninges; moreover, cellular debris at the injury site contains numerous molecules that potentially inhibit regrowth of neurons) [25], stimulate glial reactions, in principal the astrocytes' signaling up regulation of colagenous production/ gliosis (together with –including of cytoskeleton/ lipid breakdown ones – metalo–proteinases), the release/ activation of cytokines – attracting inflammatory: cells, prostaglandins (including transforming growth factor TGF-beta (TGF–beta1 and TGF–beta2 – both isoforms contributing, complementary, in the formation and respectively, in the maintenance, of glial scars) [26] and of leucotrienes – thus adding more inflammation/ cell death and consequently, fibrosis, to previous detrimental pathways in reactive/ compensatory manner: ‘fill the void (with connective, non–parenchyma, tissue) … fulfill  irreversibility’ – all resulting, including in scars – one of the major causes (aside non–replaceable loss of neurons) of the limited/ practically inefficient CNS/ SC self repair, post injury behavior (Figure 3).

To this misfortunate result, also other chemical/ physical complex inhibitory barriers for re–growth of the disrupted axons across the lesion site, seem to strongly contribute: Ephrine (EphA4), Glial Fibrillary Acidic Protein (GFAP) [1], Vimentime and respectively, the own myelin sheath.

Additionally – but basic/ intrinsic – in the CNS/ SC, from reasons yet unclear, there are  strong ‘active’ inhibitory molecules/ processes, mainly to the (spontaneous, initial post–injury tendency) of axonal re–growth and – supplementary – of CNS cells' regeneration, generically called the ‘braking’ machinery in neurons: tightly related to NOGO/(NgR (Nogo protein Receptor) proteins and more generally, to the Rho (superfamily of ‘Rho–dopsin gene – including neurotransmitter – receptors’) signaling pathway [1]; this receptors  family relays on a protein called TAJ or TROY and  on another one, p75, that act both, as important part of the same family of receptor complex proteins (called TNF receptors), on neurons, by responding to growth–inhibitory molecules in myelin and thus, preventing the cable–like axons’ (re)–growth of injured neurons in the CNS/ SC: acceptance of  these  inhibitory molecules, like a key fitting a lock and switched–on, results in inhibitory signaling, within the neuron [15]. Rho kinases (ROCKs) – the first Rho effectors to be described – are serine/ threonine kinases that are important in fundamental processes of cell: migration, proliferation and survival. Abnormal activation of the Rho/ ROCK pathway has been observed in various disorders of the CNS. Injury to the adult vertebrate brain and/or SC, activates ROCKs, thereby inhibiting neurite growth and sprouting. Conversely, the inhibition of ROCKs results in accelerated regeneration and enhanced functional recovery after SCI in mammals; moreover, inhibition of the Rho/ ROCK pathway has also proved to be efficacious in animal models of stroke, inflammatory and demyelinating diseases, Alzheimer's disease and neuropathic pain. Therefore, ROCK inhibitors, have the potential to prevent neurodegeneration and to stimulate neuroregeneration in various neurological disorders [28], thus being important, subtle targets, for neuroprotection. When these molecules bind to receptors on the surface of damaged neurons, they trigger an intracellular pathway, i.e. of the Ras – Rat sarcoma – a superfamily of (first identified as transforming onco)genes, comprising of over 150 human members [29] and divided into eight main families, each of which is further divided into subfamilies: Ras, Rho, Rab, Rap, Arf, Ran, Rheb, Rad and Rit. Miro (a recent contributor to the superfamily) – homolog gene family, member A: (RhoA)–Rho–associated, coiled–coil containing protein kinase (ROCK) pathway – that irreversibly inhibits neurite sprouting and regeneration [25].  

**Figure 4:**
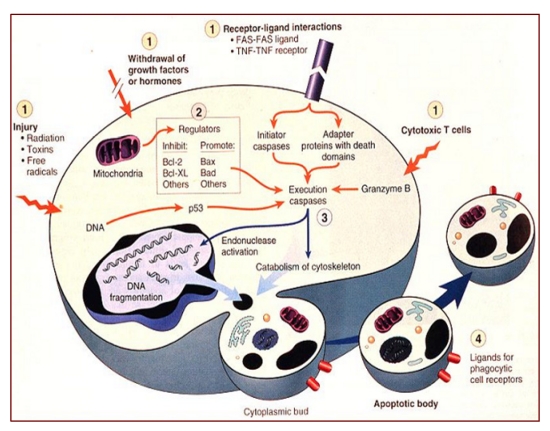
The caspases pathway of apoptosis – cited by [30]

**Figure 5:**
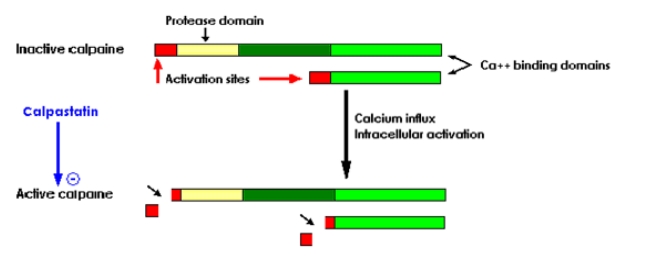
Activation of calpains – within the calpains pathway of apoptosis

**Figure 6:**
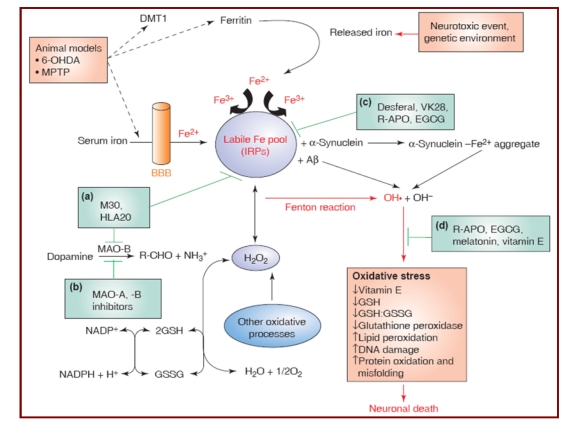
Factors and mechanism, stimulating and inhibiting, oxidative stress - by [31]

Regarding the apoptosis (Figure 4,Figure 5,Figure 6), including for neurons, the growing amount of knowledge concerning its subtle processess (especially sequential activation of key–role genes: p53, Bax, Endonuclease G – EndoG and its relation with the oxidative stress), it is mandatory – including for allowing some new both, conceptual and practical advances – to more precisely know and consequently, therapeutically target, the crucial end points within such of these pathways.

As above evoked (in relation to apoptosis) another subtle complex – and major, as well – of determinants (including) for post SCI reaction pathways, are the gene expression related changes – objectified by spatial and temporal profiling.

Therefore, micro array technology was used to identify functional groups of genes that are differentially expressed/ regulated, following contusive injury of adult rat spinal cord, at 3 h, 24 h, 7 days and 35 days; there were thus identified, including those associated with: apoptosis, cell cycle, inflammation and cholesterol metabolism. This endeavor could lead to identification of novel potential therapeutic targets within each group of examined genes [32].

In a suggestive/ metaphoric statement, the figurative linkage of events in SCI can be synthesized as follows:

- brutal aggression – ‘illegitimate occupation of territory’ – by the direct trauma/ primary injury
- ‘terror by the occupation troops, upon civil (i.e.: initially non–affected, by the primary injury, tissues) population’ – of the secondary lesions
- ‘guerrilla resistance/ sabotage’ – against  natural regeneration – of the inner (also natural) inhibiting factors.

Hence, regarding prognosis, the secondary injuries are the cross road between regaining functional status and falling into severe, (life–) long–term disability.

Therefore, trying to aim as many as possible of the afore mentioned  targets – secondary injuries and their subsequent disastrous consequences – modern research, including experimental approaches in SCI, focus on several intricate, rather overlapping, therapeutic objectives and means/ interventions, such as: neuroprotective, neurotrophic, neurorestorative, neuroreparative, neuroregenerative, neuro(re)constructive and  neurogenetic. The first three of these therapeutic directions are generically assimilated as neuroprotective [33,34].

## Integrative emphases – clinical/ therapeutic connections

In our article reviewing the main published reports on neuroprotection in SCI, we have presented a synthetically/ systematized, yet quite exhaustive, list (and related  details/ comments) of therapeutic means – pharmacological and/or and procedures – considered to have, more or less, neuroprotective properties in spinal cord and brain injuries, within a ‘color code’ [1].

Herein, we stress upon some practical suggestions to approach this subject matter, emphasizing the most, to date, efficient – obviously, within major limits, according to the afore mentioned very low capacities of CNS/ SC to self preserve and recover – and accessible drugs, respectively those that are already in clinical use (but at present, mainly to treat other – including also neurological – conditions) and therefore having relatively well known, determined, effects and/or restrictions.

**Figure 7:**
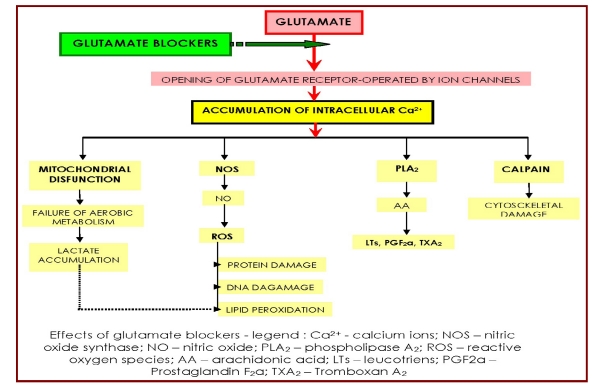
Schematic synthesis of glutamate main effects – and consequently, of its blockers

In this respect, there are to be mentioned [1]:

- anti–inflammatory/ immune modulators, including: steroid anti–inflammatory drugs (SAIDs), non– steroid anti–inflammatory drugs (NSAIDs), piritinol
- neurotropic vitamins and other nutritional supplements (B vitamins, tioctic acid, selenium, zinc, magnesium – including for the last one's non–competitive anti–NMDA receptors action), lithium (also with anti–inflammatory actions – reduces microglia and macrophage activation – by affecting the post transplantation of neural progenitor cells (NPCs), host immune response, in SCI adult rats) [35]
- anti–oxidants/ free radical scavengers: beta–carotene, ascorbate, alpha–tocopherol/ Triovit (A,C,E,), superoxide dismutase, melatonin, Q10 co–enzyme
- iron chelators: deferoxamine/ Desferal (only alternatively and if there are no other non/ contraindications and/or restrictions/ risks, in the specific patient's case); quercetin – including with cell cycle modulating effects, in combination with resveratrol, a syrtuin gene diacetilase –: Longevinex, Zen Tonic (the latter is the only drug of this kind inner marketed)
- antagonists – directly or indirectly – of growth inhibitory signals and/or of cellular energy collapse (including post injury) in CNS/ SC: statins, , phospho–diesterase inhibitors (methilxantines – theophiline tabletted medicines/ black, common, tea [36], used for hydratation, anyway –, Rolipram – used in the past, as an antidepressant – and respectively, Dibutyril cyclic AMP (the last two drugs – not yet inner marketed)
- acrolein antagonists: Acetylcysteine/ N–acetylcysteine (ACC, Fluimucil, etc.), mainly a mucolytic agent – synergistic, but by different mechanisms, with theophiline, on improving bronchial air flow – is usually necessary/ administrated in post SCI patients, in acute, sub–acute and/or sub–/hyper–chronic [37] stages (mainly in tetraplegics, that often have breathing troubles, due to the high level of the neurologic impairment, but in some of the paraplegics, too – especially in the first weeks/ months after spine surgery, when they have to keep strictly a declive position). ACC acts, on one hand, as an antioxidant, by augmenting the glutathione reserves and on the other, like an  antidote drug, counteracting acrolein, similarly with mesnum (Mesna) – likewise for their (also) common mucolytic effect [38,39]
- neurite outgrowth promoters (nucleosides: Leteprinim, Keltican/ Nucleo forte – not yet inner marketed)
- substances with neurotrophic actions: peptide mixtures (Cerebrolysin, Actovegin) and more recently, a chemically simple one, Lhitium (chloride, although carbonate is its more available for current clinical use, chemical/ pharmacological conditioning) has been shown to exert robust neuroprotective effects: stimulator of neurotrophic factors discharge (significantly increase the Nerve Growth Factor – NGF – concentrations, but only in some parts of CNS – especially in the brain) [40]; neural stem cells protector/ differentiation promoter  (the treatment of, including transplanted [35], NPCs with lithium – also a specific inhibitor of glycogen synthase kinase 3beta/   GSK3beta – aside its afore mentioned anti–inflammatory effect, significantly suppresses apoptosis of NPCs) [41]; elicitor of NPCs' differentiation/ increased neurogenesis facilitation [42] in CNS/ SC – through both, resembling mechanisms and unevenness manner (systemic application of the GSK–3 inhibitor: lithium to spinal cord–lesioned rats, suppresses the activity of this kinase, around lesion and induces significant descending corticospinal and serotonergic axon sprouting, in caudal spinal cord and promote locomotor functional recovery, GSK–3 signal thus being an important therapeutic target for promoting functional recovery of adult CNS injuries) [43]. All of these relatively recent traced actions – apart/ different from lithium's well known mood–stabilizing effects in humans – are neurotrophin ‘like’, i.e. of pleiotropic/ (even) multimodal kind – but for the moment, lithium administration in SCI should be considered only alternatively and if there are no other non/ contraindications and/or restrictions/ risks, in the specific patient's case (see further)
- apoptosis inhibitors: Minocycline (not inner marketed), Erythropoietin/ rHuEPO (only alternatively and if there are no other non/ contraindications and/or restrictions/ risks, in the specific patient's case) or – seemingly with less risk for administration – Erythropoietin carbamylate (not yet marketed)
- anti–excitotoxic agents (glutamate blockers) – Figure 7: of ionotropic receptors – NMDA (N–methyl–D–aspartic acid) – competitive (Selfotel) –, non–competitive (Memantine/ Ebixa, Amantadine, Dextropophan – not yet inner marketed –, but mild, better/ to be chosen for neuroprotection post SCI: Cerebrolysin, Magnesium)
- AMPA (alpha–amino–3–hydroxyl and #x2013;5–methyl–4–isoxazole–propionate) non–competitive (Phenytoine – only alternatively and if there are no other non/ contraindications and/or restrictions/ risks, in the specific patient's case)
- riluzole, a glutamate release inhibitor/  sodium channel blocker, with antiexcitotoxic effects, – already registered for the treatment of amiotrophic lateral sclerosis – showed, in rats: significant improvements of neuronal sparing/  survival and reinnervating capacity, after SCI, of injured motoneurons, not only when the treatment started immediately after injury but also in cases when riluzole administration was delayed for up to 10 days [44] and also was found to improve mitochondrial function, with enhancement of glutamate and glucose uptake (Methil Prednisolone – MP – treatment was found to reduce lipid peroxidation, but also improved glutamate and glucose uptake, the treatment combination: Riluzole plus MP was found to be effective in improving all five measures of oxidative stress in SCI rats – contusion type) [45]; in rabbits, riluzole was found to prevent neuronal necrosis and apoptosis and cytoskeletal proteolysis and also, to really and consistently be able to prevent SC damaging, after experimental induced SC ischemia [46]; in humans, there are yet, insufficient controlled trials to support, based on statistically significant and reliable data, the numerous and very important beneficial effects – within an integrative perspective, with combined neuroprotective strategies and approaches [47] – of this drug with seemingly, overall neuroprotective/ (even multimodal ? – o. n.) e\ctions; therefore, a group of researchers have recently (July 2009) started a multi–centre study (USA, Canada) concerning: ‘Safety of Riluzole in Patients With Acute Spinal Cord Injury’ (estimated enrollment: 36 acute SCI patients – less than 12 hours since injury –; study start date: July 2009 – but the study is not yet open for participant recruitment, last updated: July 6, 2009 –; estimated primary study completion date: April 2011 – final data collection date for primary outcome measure –; estimated study completion date: October 2011) [48]. Hence, considering, on the other hand, its possible important side effects (especially neutropenia and/or liver toxicity) we consider it preferable to wait for some serious clinical trials to be carried on, previous to administrate riluzole in SCI affected individuals (actually, this is a permanent, general problem, regarding introduction of a novel therapy in supra/ acute stages of SCI: psychologically, the patients accept it rather difficult, as they still strongly hope in a vigorous spontaneous recovery and – even doctors – can not be sure, in many cases, if administration of a new drug couldn't do more harm to the, SCI evolution, than help it, or simply, just to have any effect at all)
- (possibly/ limited indication) neuro–restoratives agents: 4–Aminopyridine/ Fampridine – a relatively selective blocker of voltage–activated potassium channels, which has been studied for its capacity to restore conduction in injured nerves including after SCI – not yet inner marketed
- calcium channel blockers (the rationale for their, hopefully neuroprotective, use during the acute phase of SCI was, on one hand – directly – to lower the intracellular calcium enhanced influx/ accumulation and on the other – indirectly – to increase micro–vascular permeability, thus diminishing ischemia and edema; but, the most serious limitation of the calcium channel blockers use in SCI, is represented exactly by the arterial hypotension – afore mentioned, ischemia generating, with its redoubtable complications – they produce, which counteracts the anticipated effect of increasing the SC blood flow): nimodipine – a voltage–operated ‘L’ channel blocker – has provided, after its administration in studies on animal models, in the acute and chronic phases of SCI, various – mostly disappointing – conclusions, i.e. while some authors found it to decrease axonal morphological damage [49] and to improve SC conduction, most researchers found no beneficial effects of this therapy [50,51].

## Discussions

**Figure 8:**
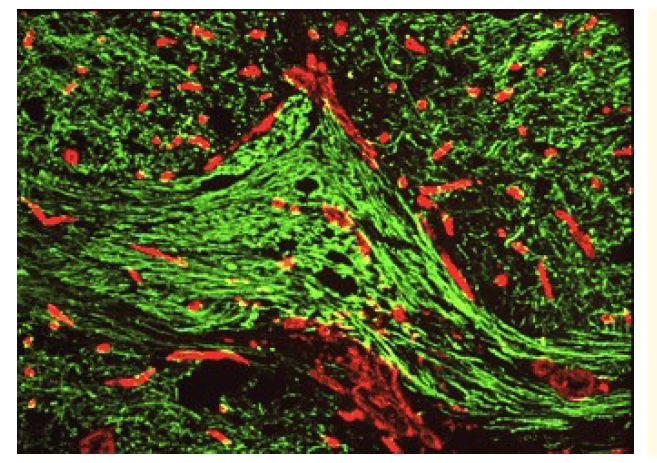
After RPT nerves are continuous - green: nerves; red: scar – by [55]

Aside the conceptual and general context/ trend related considerations, already exposed within the discussion part of the summary, herein we add some practical – arising, including from an own consistent experience of integrating/ confronting scientific highest standards/ rigors with daily clinical practice and human/ natural pressure, mainly from the patients ‘and the people who love them …’ [19] –  comments:

- there are not yet enough precise investigational data regarding all the above presented drugs' pharmaco–dynamic behavior, including their capability/ amount of  crossing the hemato–nevraxial barrier and hence, their real therapeutic contributivity to the clinical complex approach of SC/ CNS injuries (we consider this subject matter to be a further research item on our agenda, although rather difficult to scientically/ methodologically be approached) is unclear, from an Evidence Based Medicine (EBM) prospective. Moreover, the ability of a certain drug to cross the hemato–nevraxial barrier and to be effective in some CNS disturbances/ clinical conditions, does not automatically give it the possibility to exert true therapeutic effects in SCI, too: the quite intense (especially in the recent years – see above) studied lithium, for the many pathways it is targeting, with very important beneficial effects – but identified only in cell cultures and animal models, not in the clinical use – although it can enter including the CNS, this drug hasn’t shown significant results in humans; as a matter of fact, according to a very recent and rigorous trial [52], lithium, systematically administrated up to 6 months, has provided no improvement in chronic post SCI cases, except only for neuropathic pain – in patients having such symptoms (probably this action is mainly based on its classic, antidepressant properties)
  Regarding the afore given for guidance drugs – in clinical use – for acute, sub–acute and/or sub–/hyper–chronic post SCI cases, within combined formula(e), in purpose to provide as efficient as possible, pleiotropic/ (even) multimodal effects – it exist opinions according to which, as long there are not enough reliable controlled trials (for many/ most of them) to objectively prove their are effective, their use should be discouraged; strictly, from the afore evoked EBM prospective, such an attitude seems right; but, until all the huge number of medicines in clinical use nowadays, will be evaluated, each one through several controlled and rigorous trials – a hypothetic and practically impossible to be fully achieved, in a predictable future, desideratum – SCI patients appear every day and desperately ask for solution(s) to their tragedy; under these circumstances/ generally moral–deontological pressure, what should professionals choose to do – taking into account that such, afore exposed, modern therapeutic options, are drugs already tested for safety (otherwise they could not be legally allowed on the pharmaceutical market) and – at least partially – for efficacy, too and furthermore, that  many of them (increasing in number, lately) has been (dis)graded to the OTC (Over – The – Counter) category, i.e. according to rules, they are available on the pharmaceutical market even without a special medical recommendation (as it is well known: this doesn't mean they are useless, but they have relatively well determined both, beneficial actions and respectively, mainly – limited – adverse effects/ risks/ restrictions, administration related).
  Our many clinical observations/ expertise in this domain, allow the ascertainment that, although not spectacular – especially concerning motor recovery – approaching  post SCI cases by the exposed, multi–targeted pharmacological combinatory (partially individually tailored) formula, provides – aside the improvement of long–term, general health state/ survival, by, including, less complications – at least, a better compliance of such patients: it is not much but definitely is more than to act strictly through the EBM prospective, i.e. to administrate, every new SCI person almost nothing, aside supportive general therapy – if/ when needed (to say no things about his/  her  psychological reaction/ mood and further, motivation to cooperate in long–term rehabilitation programs, when told that practically, there is nothing to be done for treating SCI)
- before introducing the last comment – which is strictly medical, subject related – it is to be underlined that generally, acquiring many new(est) knowledge, of cellular/ molecular level, enables physicians to target ‘deeper’/ more intimate/ subtle pathological layers, thus optimizing different existing therapeutic substances/ agents' administration, by intelligently adding to their use (sometimes surprisingly different/ wider, respectively common) new/ (maybe even) unexpected, useful effects, beyond ‘classical’ indications – to be, at present, found in a drug leaflet or another
- although, as emphasized, to solve the tough problem of gliosis/ scars would be critical to achieve major therapeutic results, unfortunately, at present, there are no drugs/ (other) medical procedures – i.e. scar inhibitors – with such effective actions: the long awaited ‘Regeneration (? – o. n.) promoting treatment’ (RPT) of patients with acute traumatic SCI, based on Cordaneurin: a new drug, focused on preventing scar formation in acute CNS/ SC – contusive model – damage (up to 3 days post injury), is still pending. This substance, expected to revolutionize (in preclinical tests, apparently there have been obtained promising results – Figure 8) therapy in acute SCI, has yet granted, since 2004, Orphan Drug Designation by the European Medicines Agency (EMEA) for the indication: ‘treatment of traumatic spinal cord injuries’; but, although Cordaneurin was initially scheduled to enter clinical trials in 2006 and to be launched on the market in 2008, at present, there seem to be still ongoing (2009/ 2010) clinical studies on it (… ?) and this product is still non–marketed. Aside Cordaneurin, the same biotechnological producer, claims possible achievement, in the relatively near future, of a more complex (and hopefully efficient ?) treatment: Cordaneurin in combination with Stromal cell–Derived Factor–1–gamma (SDF–1gamma); this chemokine is one of the key modulators in the development of CNS: SDF–1gamma is a special type of immune modulator that (also) blocks substances which inhibit axonal (re)growth, thus acting for neuronal growth promotion – after, SCI; therefore, it seems to be strong evidence that chemokine SDF–1gamma could be a synergistic drug for the treatment with Cordaneurin (? o. n.) [53,54]

## Conclusions

Understanding deeper and deeper, both: intimate basis of the especial – and unfortunately very unfriendly – way CNS, including SC (even worse, as the latter has considerably fewer redundant cellular and connection availabilities – thus possibilities of ‘compensation by taking over’ after aggressions) and respectively the mode different therapies are targeting,  more or less effectively, some of these numerous and very complex pathways, on which relies the disastrous post injury consequences, has provided the specialists dealing with SCI, better instruments for neuroprotection and even more – for pleiotropic/ multimodal effects.
